# Non-Invasive Monitoring for Rejection in Kidney Transplant Recipients After SARS-CoV-2 mRNA Vaccination

**DOI:** 10.3389/fimmu.2022.838985

**Published:** 2022-02-25

**Authors:** Ayman Al Jurdi, Rodrigo B. Gassen, Thiago J. Borges, Zhabiz Solhjou, Frank E. Hullekes, Isadora T. Lape, Orhan Efe, Areej Alghamdi, Poojan Patel, John Y. Choi, Mostafa T. Mohammed, Brigid Bohan, Vikram Pattanayak, Ivy Rosales, Paolo Cravedi, Camille N. Kotton, Jamil R. Azzi, Leonardo V. Riella

**Affiliations:** ^1^ Center for Transplantation Sciences, Massachusetts General Hospital, Boston, MA, United States; ^2^ Transplantation Research Center, Renal Division, Brigham and Women’s Hospital, Boston, MA, United States; ^3^ Clinical Pathology Department, Minia University, Minya, Egypt; ^4^ Histocompatibility Laboratory, Massachusetts General Hospital, Boston, MA, United States; ^5^ Department of Pathology, Massachusetts General Hospital, Boston, MA, United States; ^6^ Renal Division, Department of Medicine, Icahn School of Medicine at Mount Sinai, New York, NY, United States; ^7^ Transplant and Immunocompromised Host Infectious Diseases Infectious Diseases Division, Massachusetts General Hospital, Harvard Medical School, Boston, MA, United States

**Keywords:** SARS-CoV-2, COVID-19, kidney transplant, vaccine, rejection, monitoring

## Abstract

**Introduction:**

Studies have shown reduced antiviral responses in kidney transplant recipients (KTRs) following SARS-CoV-2 mRNA vaccination, but data on post-vaccination alloimmune responses and antiviral responses against the Delta (B.1.617.2) variant are limited.

**Materials and methods:**

To address this issue, we conducted a prospective, multi-center study of 58 adult KTRs receiving mRNA-BNT162b2 or mRNA-1273 vaccines. We used multiple complementary non-invasive biomarkers for rejection monitoring including serum creatinine, proteinuria, donor-derived cell-free DNA, peripheral blood gene expression profile (PBGEP), urinary *CXCL9* mRNA and *de novo* donor-specific antibodies (DSA). Secondary outcomes included development of anti-viral immune responses against the wild-type and Delta variant of SARS-CoV-2.

**Results:**

At a median of 85 days, no KTRs developed *de novo* DSAs and only one patient developed acute rejection following recent conversion to belatacept, which was associated with increased creatinine and urinary *CXCL9* levels. During follow-up, there were no significant changes in proteinuria, donor-derived cell-free DNA levels or PBGEP. 36% of KTRs in our cohort developed anti-wild-type spike antibodies, 75% and 55% of whom had neutralizing responses against wild-type and Delta variants respectively. A cellular response against wild-type S1, measured by interferon-γ-ELISpot assay, developed in 38% of KTRs. Cellular responses did not differ in KTRs with or without antibody responses.

**Conclusions:**

SARS-CoV-2 mRNA vaccination in KTRs did not elicit a significant alloimmune response. About half of KTRs who develop anti-wild-type spike antibodies after two mRNA vaccine doses have neutralizing responses against the Delta variant. There was no association between anti-viral humoral and cellular responses.

## Introduction

Coronavirus disease (COVID-19) caused by severe acute respiratory virus coronavirus 2 (SARS-CoV-2) is associated with increased mortality in kidney transplant recipients (KTRs) compared to non-transplant patients (1). Despite studies showing a blunted antibody response (2–4), SARS-CoV-2 vaccination has been associated with reduced incidence of COVID-19 and reduced case-fatality rate in KTRs (5–7). Reports of anti-viral humoral and cellular responses in KTRs following SARS-CoV-2 messenger RNA (mRNA) vaccination have been published (2–4, 8–10), but they were focused on Wild-type SARS-CoV-2 with very little data on antiviral responses against the Delta (B.1.617.2) variant (11), which accounts for most infections at this time in the United States (12).

Furthermore, data about the monitoring of alloimmune responses and graft function in KTRs following SARS-CoV-2 mRNA vaccination remain limited (13–16). Concerns about the use of mRNA vaccines in KTRs include their excessive activation of the immune system and their potential for triggering allograft rejection (17–19). The concern stems at least partially from that SARS-CoV-2 mRNA vaccines have been shown to elicit a strong cytokine response in CD4^+^ T-cells from immunocompetent individuals, including IL-2 and TNF-α (20). Furthermore, kidney allograft rejection has been reported following SARS-CoV-2 mRNA vaccination (14). Assessment of alloimmune responses to these vaccines is crucial to provide guidance regarding the need for more frequent monitoring for rejection following vaccination in KTRs. Therefore, it is of paramount importance to conduct a comprehensive evaluation of allograft status by complementary methods and assess alloimmune responses following SARS-CoV-2 mRNA vaccination in KTRs.

The aim of this study was to comprehensively monitor allograft status using several non-invasive additional tools beyond what is routinely done clinically and what has been done in other studies. We also characterized the alloimmune and anti-viral responses after SARS-CoV-2 mRNA vaccination in KTRs, including antiviral responses against the Delta variant.

## Materials and Methods

### Study Design and Patient Recruitment

**Figure 1:**
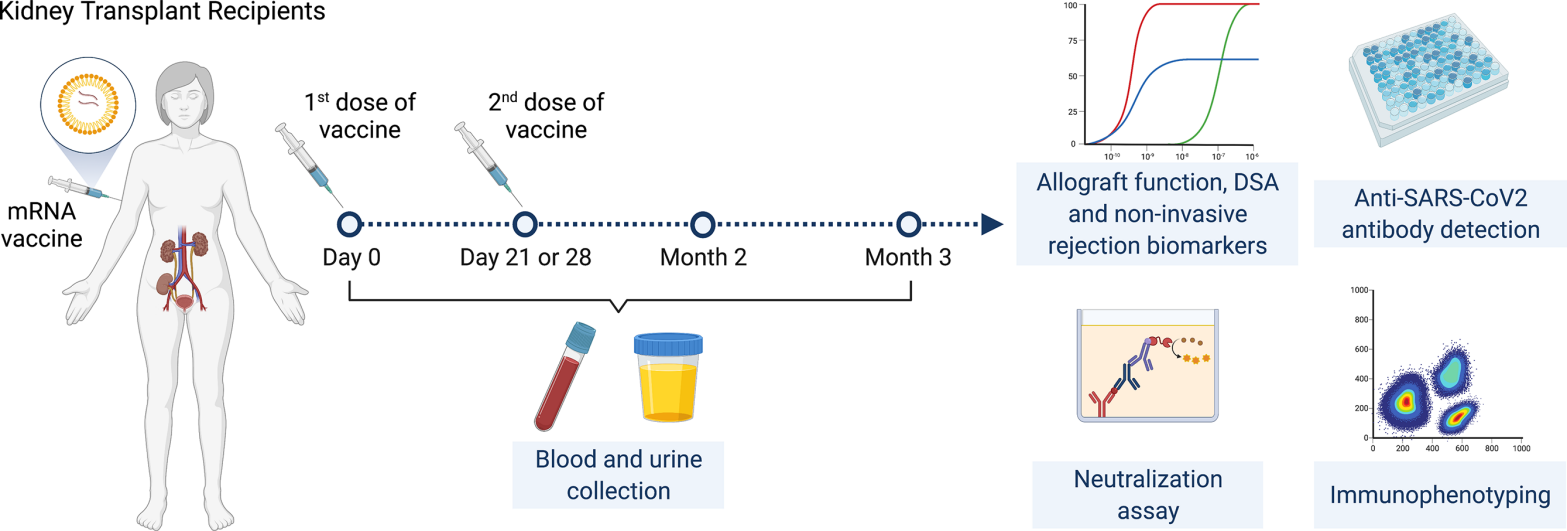
Study design.

This is a prospective multicenter observational cohort study of the safety and efficacy of SARS-CoV-2 mRNA vaccines in adult KTRs (**Figure 1**). Inclusion criteria were KTRs >3 months post-transplantation who were ≥18 years of age with stable allograft function (<20% variation in last two eGFR values at least one week apart) and no rejection in the prior six months. KTRs were enrolled consecutively by order of vaccination. Full exclusion criteria are listed in supplementary material and methods.

Enrolled KTRs received two doses of SARS-CoV-2 mRNA vaccines administered 28 days apart for the mRNA-1273 vaccine and 21 days apart for the BNT162b2 vaccine. Participants had a study visit at baseline (day 0, immediately prior to first vaccine dose) then had follow-up visits immediately prior to the second vaccine dose (day 21 or 28 depending on vaccine), at two months and at three months following initial vaccination. Baseline characteristics were reviewed at the initial visit and adverse events were reviewed at follow-up visits. Blood and urine samples were collected at all timepoints.

### Study Approval

The study was approved by the institutional review board at Mass General Brigham (Protocol#: 2021P000043). All subjects signed written informed consent forms prior to inclusion in the study. The clinical and research activities being reported are in compliance with the Declaration of Helsinki and consistent with the Principles of the Declaration of Istanbul. Data are reported in accordance with the STROBE statement reporting guidelines.

### Outcomes

The primary objective was non-invasive rejection detection using a combination of biomarkers including serum creatinine, urine protein-to-creatinine ratio (UPCR), *de novo* donor-specific antibodies (DSAs), donor-derived cell-free DNA (ddcfDNA), peripheral blood gene expression profile (PBGEP) and urinary *CXCL9* mRNA. Secondary outcomes included 1) generation of anti- spike antibodies against wild-type and Delta variant of SARS-CoV-2 (assessed by a Luminex-based multiplex assay and a surrogate virus neutralization test [SVNT]); 2) S1-specific cellular immunity against wild-type SARS-CoV-2 (assessed by an IFN-γ EliSPOT assay and immune phenotyping using flow cytometry), 3) development of any severe or grade 4 adverse events and 4) development of SARS-CoV-2 infection within three months of vaccination.

### Sample Collection and Processing

Blood and urine samples were collected from KTRs at baseline, prior to the second vaccine dose, and at two- and three-months post-vaccination. Blood and urine samples were sent to the clinical lab (for serum creatinine and urine protein-to-creatinine ratio quantification), our research laboratories (for DSA, anti-spike antibody and cellular immune assays) and CareDx, Inc. (for ddcfDNA and PBGEP).

Serum and plasma were obtained from peripheral blood by centrifuging for 15 minutes at 2,500 RPM at room temperature then stored in cryogenic tubes at -80°C. PBMCs were isolated using SepMate™-50 tubes (Stemcell technologies) containing Lymphoprep™ solution (Stemcell technologies) then centrifuged at 1,200g for 10 minutes. The PBMC layer was then transferred to a new 50 mL conical tube and centrifuged at 300g for 8 minutes. The supernatant was discarded, and the pelleted cells were resuspended in 10ml of sterile PBS 1x then centrifuged again at 300g for 8 minutes. Pelleted cells were re-suspended in a solution of GemCell™ human serum (GeminiBio) with 10% of dimethyl sulfoxide (100μL for each 1 million PBMCs), aliquoted in cryogenic tubes then stored at -80°C for 1-3 days before being transferred to a liquid nitrogen freezer.

### Donor-Derived Cell-Free DNA Assay

Circulating ddcfDNA levels were measured as per published AlloSure^®^ protocol (CareDx, Inc., Brisbane, CA) (21, 22). Briefly, duplicate samples of venous blood were collected using Streck cell-free DNA BCT^®^ tubes then shipped to CareDx, Inc. laboratories (Brisbane, CA), where plasma was separated by centrifugation then cell-free DNA was extracted using the circulating nucleic acid kit (Qiagen, cat no. 55114) following manufacturer’s instructions. Plasma ddcfDNA levels were measured using a next-generation sequencing assay utilizing 266 single nucleotide polymorphisms, which allows quantification of ddcfDNA without requiring genotyping of the donor or recipient. Results are reported as percentage of total circulating cell-free DNA. Percentages above 0.5-1.0% are associated with an increased risk of allograft rejection (22, 23). Fifty one out of fifty eight patients had Streck cell-free DNA BCT^®^ tubes collected for ddcfDNA measurement.

### Peripheral Blood Gene Expression Profile (PBGEP) Assay

PBGEP assay was performed and PBGEP score calculated as per AlloMAP protocol (CareDx, Inc., Brisbane, CA) (24). As described previously (24), peripheral blood was collected in PAXgene^®^ RNA tubes then shipped to CareDx, Inc. laboratories (Brisbane, CA) where RNA was extracted using the QIASymphony PAXgene Blood RNA kit (Qiagen, Cat no. 762635) following manufacturer’s instructions. QIASymphony spectrophotometry system (Qiagen, Hilden, Germany) was used to determine the concentration and purity of RNA. Quantitative real-time reverse transcriptase polymerase chain reaction (qRT-PCR) was run in triplicates for purified RNA samples and the mean C_T_ for each determined as described previously (25). The mean C_T_ for the five candidate genes (DCAF12, MARCH8, FLT3, IL1R2, and PDCD1) derived from previous studies in heart transplantation (26), was normalized to six reference genes (24). A multivariate model that integrates reference-normalized expression of the five genes computes a PBGEP (AlloMAP-Kidney) score (range 0-20) to differentiate immune quiescence from rejection with higher scores associated with rejection. Median (IQR) scores of 12.43 (11.12-14.29) and 10.19 (7.64-12.09) were seen in patients with and without kidney allograft rejection respectively (24). Fifty-one out of fifty-eight patients had PAXgene^®^ RNA tubes collected for PBGEP scoring.

### Anti-Human Leukocyte Antigen Antibody Assays

Screening for anti-human leukocyte antigen (HLA) antibodies was performed at 3 months post-vaccination using mixed class I & II kit (One Lambda, catalog no. LSM12). Briefly, patient sera and negative control sera (One Lambda, catalog no. LS-NC) were added to HLA-coated beads and incubated for 30 minutes at room temperature. After washing, PE-conjugated goat anti-human IgG secondary antibody (One Lambda, catalog no. LS-AB2) was added then incubated for 30 minutes. The results were read using LABScan™ 100 (Luminex, Austin, TX) and analyzed using HLA Fusion™ software (One Lambda). A sample to negative control serum mean fluorescent intensity (MFI) ratio >3.5 was considered positive per our histocompatibility lab’s standards. Patients with a positive anti-HLA antibody screen at month 3 then had anti-HLA antibody class I and II testing using single-antigen beads (LABScreen™ Single Antigen Class I – Combi, catalog no. LS1A04 and Class II – Group 1, catalog no. LS2A01, One Lambda) with an identical protocol to determine which anti-HLA antibodies were present to determine if they were donor-specific. Baseline (pre-vaccination) sera were then tested to determine if DSAs had been present prior to vaccination or were *de novo* (defined as new DSAs with MFI >1,000). In patients with pre-existing DSAs, we also evaluated increases in the MFIs of pre-existing DSAs following vaccination, which we defined as a 50% increase in MFI from pre-vaccination baseline.

### Urinary *CXCL9* mRNA Measurement by CRISPR-Cas13 Platform

Urinary *CXCL9* measurement was performed in 26 KTRs recruited at one of the transplant sites, where urine pellets had been processed prior to and following vaccination. Briefly, 45ml of urine were collected from patients then centrifuged at 2,000g for 30 minutes at 4°C. The supernatant was discarded, the pellet was washed in 1ml of PBS and then centrifuged in a microcentrifuge tube at 10,000g for 5 minutes at 4°C. The supernatant was discarded and 200μL of RNAlater^®^ (QIAGEN, cat no. 76163) were added and stored at -80°C. RNA was isolated using RNeasy Micro Kit (Qiagen, catalog no. 55114) following manufacturer instructions. Recombinase polymerase amplification (RPA) and Cas-13 reactions were performed in duplicates as previously described by our group (27). The reaction was read using SpectraMax iD3 microplate reader (Molecular Devices, San Jose, CA). Net relative fluorescent units (RFU) values were determined by subtraction from background values. A cut-off of 100 background-subtracted RFUs was considered positive.

### Specific-Antibody Quantification and Neutralization Capacity

Total anti-SARS-CoV-2 antibodies (IgM, IgA and IgG) against the spike protein trimer, S1 region, receptor-binding domain (RBD) region and nucleocapsid (NC) protein from baseline and month two after vaccination samples were measured using the Coronavirus Ig Total Human 11-Plex ProcartaPlex™ Panel (Invitrogen™, catalog no. EPX110-16000-901). Capture beads were added to each well and then controls and samples (in a 1:1,000 dilution) were added. The plate was incubated for 2 hours at room temperature, then washed and detection antibody was added to each well. After a 30-minute incubation at room temperature, the plate was read using MAGPIX system (Luminex, Austin, TX) and the data was analyzed using xPONENT software (Luminex, Austin, TX). As indicated by the manufacturer, a positive result was defined as a sample to low-control MFI ratio above 1.3. An indeterminate result was defined as an MFI ratio between 1.0 and 1.3, while a MFI ratio <1.0 was defined as a negative result.

In patients who had a positive or indeterminate anti-SARS-CoV-2 antibody result, we evaluated the neutralizing function of antibodies against both wild-type and Delta (B.1.617.2) variant of SARS-CoV-2 using a surrogate virus neutralization test (GenScript cPass kit, catalog no. L00847-A). Patient sera (diluted 1:10) were incubated at 37°C for 30 minutes with horseradish peroxidase-conjugated recombinant SARS-CoV-2 RBD fragments (HRP-RBD) in a 1:1 ratio. The mix of sera and HRP-RBD (Delta RBD-HRP with L452R and T478K mutations, GenScript catalog no. Z03614-20) was added to each well of a capture plate pre-coated with human angiotensin converting enzyme 2 protein (ACE2) then incubated at 37°C for 15 minutes. Neutralizing antibodies form complexes with HRP-RBD that remain in the supernatant are removed with washing, while non-neutralizing antibodies-HRP-RBD complexes and unbound HRP-RBD bind to ACE2 and are captured on the plate. After washing, 3,3’,5,5’-Tetramethylbenzidine (TMB) solution was added, and the plate was incubated in the dark for 15 minutes at room temperature. Finally, a stop solution was added, and the plate was read at 450nm using SpectraMax iD3 microplate reader (Molecular Devices, San Jose, CA). The absorbance of the sample is inversely related to the concentration of neutralizing antibody. As indicated by the manufacturer, inhibition of ≥30% was considered a positive result. The concentration of neutralizing antibody was determined using a neutralizing antibody standard curve (GenScript, catalog no. A02087).

### ELISpot for IFN-γ Quantification

IFN-γ ELISPOT was performed as described previously (28). Briefly, frozen PBMCs were thawed, washed and 0.5 million cells were added to ELISpot^PLUS^ plates pre-coated with anti-IFN-γ antibodies (Mabtech, catalog no. 3420-4AST-P1-1). The cells were stimulated with SARS-CoV-2 peptides from the S1 region of the spike protein at a concentration of 2 μg/mL along with CD28-stimulating antibody at a concentration of 0.1 μg/mL for 48 hours in a humidified incubator (37°C with 5% of CO_2_). Monoclonal CD3-stimulating antibody was used as a positive control and unstimulated PBMCs were used as negative controls. The detection antibody (7-B6-1-Biotin) was then added and the plate and incubated for two hours at room temperature. Afterwards, Streptavidin-ALP and then its substrate solution (BCIP/NBT-plus) were added. The reaction was left to develop until distinct spots appeared, after which it was stopped by extensive washing with tap water. After air drying, the plate was read using KS ELISpot reader (Zeiss, Thornwood, NY) with software version KS ELISpot 4.9.16 (29). Results are reported as spots per 10^6^ PBMCs. An increase of ≥32 spots per 10^6^ PBMCs from baseline was defined as a positive result. The cut-off was determined as an increase of 3 standard deviations of the negative controls as was done in previous studies (13).

### Immune Phenotyping With Flow Cytometry

Frozen PBMCs were thawed, and one million cells were added per well in a U-bottom 96 well plate and incubated for 4 hours in a humidified incubator (37°C, 5% CO_2_). After this incubation, the cells were transferred to a V-bottom 96 well plate for the staining. One plate was used for B-cell marker staining, and the other plate was used for T-cell markers staining. B-cell antibodies used were CD19 (PE, clone 4G7, BioLegend), IgM (BUV395, clone G20-127, BD Bioscience), IgD (PerCP/Cyanine5.5, clone IgD, BioLegend), IgG (FITC, clone G18-145, BD Bioscience), CD22 (BV421™, clone S-HCL-1, BioLegend), CD24 (APC/Cyanine7, clone SN3 A5-SH10, BioLegend), CD27 (APC, clone O323, BioLegend), CD138 (BUV737, clone MI15, BD Biosciences), CD3 (BV605™, clone OKT3, BioLegend), CD5 (BV711™, clone UCHT2, BioLegend), HLA-DR (BV650™, clone L243, BioLegend), CD274/PD-L1 (BV786, clone MIH1, BD Biosciences), CD279/PD-1 (Alexa Fluor^®^, clone EH12.2H7, BioLegend), CD25 (PE/Dazzle™ 594, clone M-A251, BioLegend) and CD38 (PE/Cyanine7, clone HIT2, BioLegend).

T-cell antibodies used were anti-CD8 (BUV737, clone SK1, BD Biosciences), CD4 (BUV395, clone SK3, BD Biosciences), CD3 (BV605™, clone OKT3, BioLegend), CD25 (PE, clone M-A251, BioLegend), CD185/CXCR5 (BV711™, clone J252D4, BioLegend) CD45RA (APC, clone HI100, BioLegend), CD279/PD-1 (Alexa Fluor^®^, clone EH12.2H7, BioLegend), HLA-DR (APC/Cyanine7, clone L243, BioLegend), CD197/CCR7 (PE/Dazzle™ 594, clone G043H7, BioLegend), CD196/CCR6 (PE/Cyanine7, clone G034E3, BioLegend), CD127 (PerCP/Cyanine5.5, clone A019D5, BioLegend) and CXCR3 (FITC, clone G025H7, BioLegend). Samples were read using BD LSRFortessa™ X-20 cell analyzer (BD Biosciences, Franklin Lakes, NJ) and the data was analyzed using FlowJo 10 (FlowJo, Ashland, OR). The gating strategy can be found in **Figures S1** and **S2**.

### Statistics

Continuous variables are presented as means ( ± standard deviation) or medians (with interquartile ranges) depending on normality of distribution. Categorical variables are presented as frequencies and percentages. For continuous variables, differences between unpaired samples were assessed using an unpaired t-test or Mann-Whitney U-test depending on distribution. Differences between two paired samples were assessed using a paired t-test or Wilcoxon matched-pairs signed rank test as appropriate. Differences between paired samples at multiple follow-up timepoints were assessed using one-way repeated measures analysis of variance, Friedman test or mixed-effects model as appropriate. For tests that reached statistical significance, pairwise testing was performed to determine significant differences between the groups, using Bonferroni correction to adjust for multiple pairwise comparisons. For categorical variables, differences in proportions were calculated using Pearson’s Chi squared test or Fisher’s exact test as appropriate. The odds of developing an outcome based on an exposure variable were expressed as an odds ratio. Multivariable logistic regression was used to adjust for potential confounding co-variates in determining the odds of developing outcomes based on an exposure variable. Pearson’s or Spearman’s correlation coefficient were calculated to evaluate correlations between continuous variables as appropriate. All tests used were two-sided and a two-sided α-level of 0.05 was considered to be statistically significant. SPSS v24 (Chicago, IL) and GraphPad Prism v9.1.2 (San Diego, CA) were used for statistical analysis and creation of figures.

## Results

### Patient Characteristics

Fifty-eight KTRs were enrolled in the study. Baseline characteristics of KTRs are shown in **Table 1**. Median age was 62 years, 41% were female, median time post-transplantation was 47 months (range 4-401) and 9% had DSAs at the time of vaccination (**Table S1**). Fifty-six (97%) patients received the BNT162b2 vaccine. Patients were followed for a median of 85 days (IQR 81-88).

**Table 1:** Baseline characteristics of kidney transplant recipients.

Baseline characteristic	n = 58
Age at enrollment (years), median (IQR)	62 (51-70)
Time from transplantation (months), median (range)	47 (4-401)
Female sex, n (%)	24 (41)
Previous kidney transplant, n (%)	
None	52 (90)
One	4 (7)
Two	2 (3)
Cause of ESKD, n (%)	
Glomerular disease	21 (36)
Diabetic nephropathy	10 (17)
Polycystic kidney disease	9 (16)
Genetic kidney disease	5 (9)
Obstructive uropathy	2 (3)
Lithium toxicity	2 (3)
Other or unknown	9 (16)
Pre-transplant RRT, n (%)	
None	24 (41)
Hemodialysis	28 (48)
Peritoneal dialysis	6 (10)
Donor source, n (%)	
Living related	11 (19)
Living unrelated	24 (41)
Deceased	23 (40)
Cold ischemia time (hours), median (IQR)	8.0 (1.0-14.0)
KDPI (%), median (IQR)	46 (31-69)
HLA ABDR mismatches, median (IQR)	3 (4-5)
Class I PRA (%), median (range)	0 (0-69)
Class II PRA (%), median (range)	0 (0-97)
Pre-transplant DSA, n (%)	3 (5)
DSA at the time of vaccination, n (%)	5 (9)
Induction immunosuppression, n (%)	_
Anti-thymocyte globulin	26 (45)
Basiliximab	25 (43)
Data not available	7 (12)
Maintenance immunosuppression, n (%)	_
Calcineurin inhibitor	_
Tacrolimus	36 (62)
Trough level in ng/mL, median (IQR)	5.9 (4.6-7.2)
mTOR inhibitor	_
Everolimus	5 (9)
Sirolimus	4 (7)
Belatacept	16 (28)
Mycophenolate	45 (78)
Total daily dose in mg, median (IQR)	1,000 (1,000-1,000)
Azathioprine	5 (9)
Prednisone	43 (74)
Total daily dose in mg, median (IQR)	5 (5-5)
Rituximab (within 12 months)	1 (2)
CMV serostatus, n (%)	
D+/R+	4 (7)
D+/R-	11 (19)
D-/R+	10 (17)
D-/R-	24 (41)
Not available	9 (16)
EBV serostatus, n (%)	
D+/R+	39 (67)
D+/R-	5 (9)
D-/R+	1 (2)
D-/R-	1 (2)
Not available	12 (21)
Delayed graft function, n (%)	12 (24)
History of allograft rejection, n (%)	14 (24)
Months since most recent rejection, median (IQR)	37.7 (15.9-54.3)
Serum creatinine (mg/dL), median (IQR)	1.22 (1.06-1.66)
Estimated GFR (ml/min/1.73 m^2^), median (IQR)	57.0 (42.3-70.8)
Urine protein to creatinine ratio (g/g), median (IQR)	0.13 (0.09-0.27)
Donor-derived cell free DNA (%), median (IQR)	0.15 (0.00-0.24)
Previous SARS-CoV-2 infection, n (%)	2 (3)
mRNA vaccine received, n (%)	
mRNA-BNT162b2	56 (97)
mRNA-1273	2 (3)
ACE inhibitor or ARB use, n (%)	15 (26)

ACE, Angiotensin converting enzyme; ARB, Angiotensin receptor blocker; CMV, Cytomegalovirus; EBV, Epstein-Barr virus; ESKD, end-stage kidney disease; GFR, glomerular filtration rate; HLA, human leukocyte antigen; KDPI, kidney donor profile index; mTOR, Mammalian target of rapamycin; PRA, panel reactive antibodies; RRT, renal replacement therapy;

### Allograft Rejection, Non-Invasive Rejection Monitoring and *De Novo* DSA Generation

**Figure 2:**
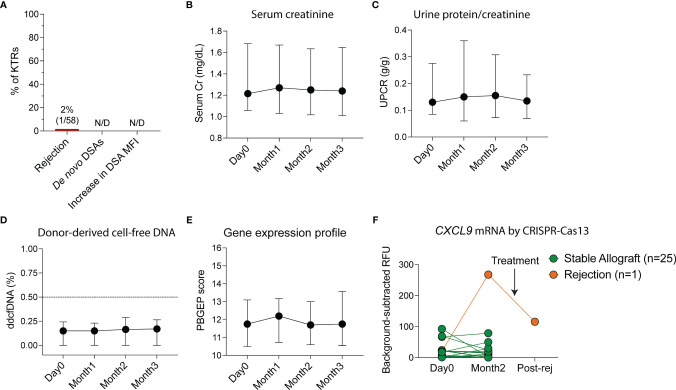
Non-invasive monitoring of allografts in kidney transplant recipients (KTRs) following SARS-CoV-2 mRNA vaccination. **(A)** Incidence of rejection, *de novo* donor-specific antibody (DSA) generation and increase in DSA mean fluorescent intensity (MFI) following vaccination (n=58). **(B)** Non-invasive monitoring for allograft rejection in KTRs with serum creatinine (Cr, n=58, p=0.292) **(C)** urine protein-creatinine ratio (UPCR, n=58, p=0.428), **(D)** donor-derived cell-free DNA (ddcfDNA, n=51, p=0.114) and **(E)** peripheral blood gene expression profile (PBGEP, n=51, p=0.393) score following vaccination. **(F)** Urine *CXCL9* mRNA relative fluorescent units (RFUs) detected by CRISPR-Cas13 in KTRs prior to and following vaccination at 4 weeks after second dose of the vaccine (n=26). **(B-E)** Medians and IQRs are shown. Statistics by mixed-effect analysis. Post-rej: post-rejection.

To monitor for rejection non-invasively, we measured serum creatinine, UPCR, *de novo* DSAs by single-antigen bead assay, ddcfDNA as a marker of graft injury (22), 5-gene rejection PBGEP (24), and urinary CXCL9 mRNA levels using a CRISPR-Cas13 platform (27). No patients developed *de novo* DSAs or a significant increase in pre-vaccination DSA mean fluorescent intensities (MFIs) at three months post-vaccination (**Figure 2A**). Only one patient developed acute cellular rejection 40 days following initial vaccination (**Figures 2A** and **S3**) in the setting of conversion from tacrolimus to belatacept two days pre-vaccination. He presented with a rise in creatinine concomitant with a rise in urinary *CXCL9* mRNA, without *de novo* DSAs and with stable proteinuria, ddcfDNA levels and PBGEP score. The remaining patients had stable serum creatinine, proteinuria, ddcfDNA levels and PBGEP scores during follow-up (**Figures 2B-E**). At two months post-vaccination, only the patient with rejection had elevated urinary *CXCL9* mRNA levels, which decreased with rejection treatment (**Figure 2F**).

### Antibody-Mediated Viral Immunity

**Figure 3:**
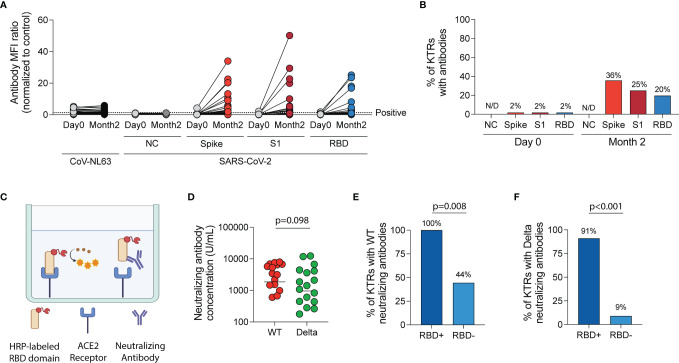
Humoral immune response following SARS-CoV-2 mRNA vaccination in kidney transplant recipients (KTRs). **(A)** Anti-SARS-CoV-2 spike antibody and anti-CoV-NL63 antibody MFI ratios (normalized to control) in KTRs pre- and post-vaccination by Luminex-based multiplex assay (n=57). **(B)** The percentage of KTRs with antibodies to SARS-CoV-2 antigens prior to and following vaccination (n=57). **(C)** Surrogate virus neutralization test diagram. **(D)** Concentration of neutralizing antibodies against Wild-type (WT) and Delta (B.1.617.2) variant of SARS-CoV-2 in KTRs with anti-spike antibodies following vaccination (horizontal line at median concentration, n=20). **(E)** Percentage of anti-spike antibody positive KTRs with neutralizing antibodies against WT and **(F)** Delta variant of SARS-CoV-2 stratified by anti-RBD antibody status (n=20). **(A, D)** Statistic by Wilcoxon matched-pairs signed rank test. **(E, F)** Statistic by Fisher’s exact test. ACE, Angiotensin-converting enzyme; HRP, horseradish peroxidase; MFI, median fluorescent intensity; NC, nucleocapsid; RBD, receptor-binding domain.

After vaccination, there was a significant increase in MFI ratios of antibodies directed against the spike trimer, S1 and RBD domains of wild-type SARS-CoV-2 (p<0.001 for all, **Figure 3A**) but no change in MFI ratios for antibodies directed against the nucleocapsid (NC) protein of wild-type SARS-CoV-2 (p=0.309) or against a control virus, CoV-NL63 (p=0.135). Using the positivity threshold recommended by the manufacturer, one patient (2%) had antibodies against the wild-type spike trimer, S1 and RBD regions of the spike protein prior to vaccination. This patient was one of two KTRs with documented prior SARS-CoV-2 infection. At the two-month timepoint after vaccination (median 55 days post-first vaccine, IQR 49-58), no KTRs had anti-NC antibodies but 36%, 25% and 20% had a positive result for antibodies against the spike trimer, S1 and RBD regions of wild-type SARS-CoV-2, respectively (**Figure 3B**).

In KTRs with a positive anti-wild-type spike antibody result, surrogate virus neutralization assay (**Figure 3C**) showed neutralizing responses in 75% and 55% against wild-type and the Delta variant, respectively (p=0.185). The median concentration of neutralizing antibodies was 2,179 U/mL (IQR 638-6,116 U/mL) against wild-type SARS-CoV-2 and 955 U/mL (IQR 278-3,750 U/mL) against the Delta variant (p=0.098, **Figure 3D**). KTRs who had both anti-spike and anti-RBD antibodies were more likely to have a neutralizing response against wild-type (RR=2.3 [95% CI:1.1-4.7], **Figure 3E**) and Delta variant (RR=9.8 [95% CI: 2.2-55.3], **Figure 3F**) of SARS-CoV-2 compared to KTRs who had anti-spike but not anti-RBD antibodies.

**Figure 4:**
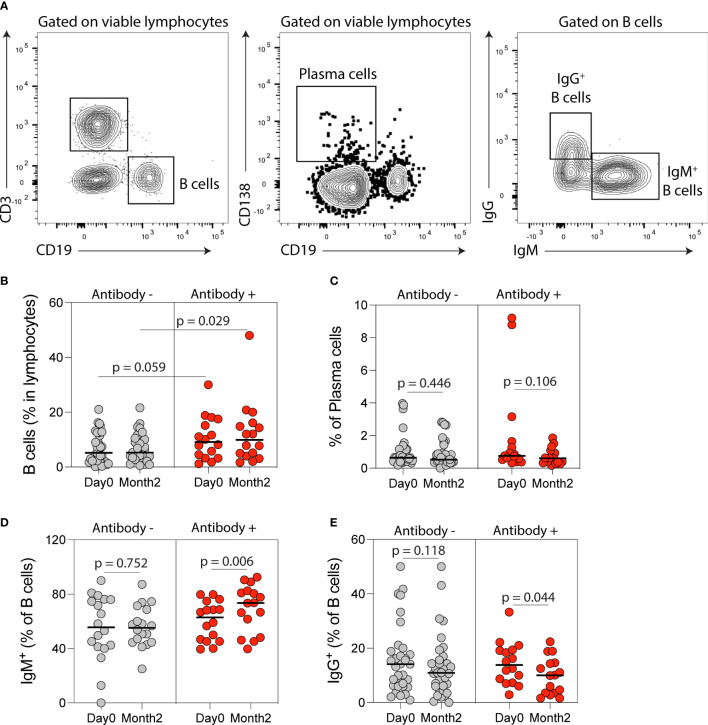
B cell immune phenotyping following SARS-CoV-2 mRNA vaccination in kidney transplant recipients (KTRs). **(A)** Flow cytometry gating strategy of B-cells, plasma, IgM- and IgG-producing B-cells. Percentages of circulating **(B)** B-cells, **(C)** plasma cells, **(D)** IgM^+^ B-cells and **(E)** IgG^+^ B-cells after vaccination in KTRs who developed anti-spike antibodies (Antibody+) compared to KTRs who did not develop anti-spike antibodies (Antibody-) (n=49). **(B)** Statistic by unpaired t test. **(C-E)** Statistics by paired t test. Horizontal lines represent mean values.

Flow cytometry analysis of circulating B-cell populations (**Figures 4A** and **S1**) showed an increase in the percentage of IgM^+^ B-cells and a decrease in the percentage of IgG^+^ B-cells post-vaccination (**Figures S4A-N**). When analysis was stratified by anti-spike antibody response post-vaccination, the percentage of B-cells was higher at month two in antibody-positive compared to antibody-negative KTRs (*p*=0.029, **Figure 4B**) and there was a trend towards a higher B-cell percentage at baseline in antibody-positive KTRs (*p*=0.059, **Figure 4B**). There was no difference in the percentage of plasma cells in antibody-positive compared to antibody-negative KTRs pre- or post-vaccination (**Figure 4C**). There was a significant increase in the percentage of IgM^+^ B-cells (*p*=0.006, **Figure 4D**) and a decrease in the percentage of IgG^+^ B-cells post-vaccination in antibody-positive KTRs (*p*=0.044, **Figure 4E**). There was also a significant increase in the percentage of IgM^+^ plasma cells in antibody-positive KTRs post-vaccination (*p*=0.008, **Figure S4O**) but no change in the percentage of IgG^+^ plasma cells post-vaccination in either group (**Figure S4P**).

### Cellular Immune Response Following SARS-CoV-2 Vaccination

**Figure 5:**
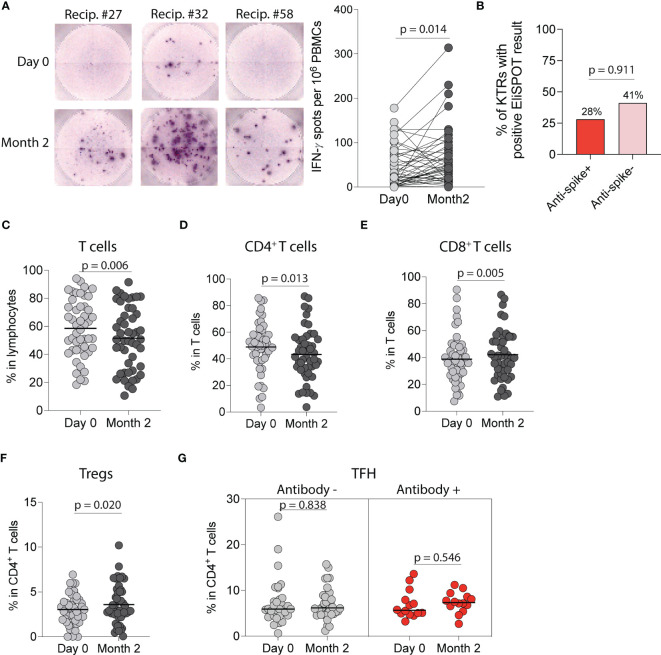
Cellular immune response following SARS-CoV-2 mRNA vaccination in kidney transplant recipients (KTRs). **(A)** EliSPOT assay for IFN-γ production by PBMCs from KTRs incubated with SARS-CoV-2 S1 peptides following vaccination (n=53). **(B)** Percentage of ELISpot response in KTRs stratified by anti-spike antibody status (n=53). Percentage of circulating **(C)** T-cells, **(D)** CD4^+^ T-cells, **(E)** CD8^+^ T-cells, and **(F)** CD4^+^ regulatory T-cells (Tregs) in KTRs following vaccination (n=49). **(G)** Percentage of circulating T follicular helper (TFH) cells in KTRs with and without anti-spike antibodies (Antibody + and -, respectively) following vaccination (n=49). **(A, C-G)** Statistic by Wilcoxon matched-pairs signed rank test. Horizontal lines represent median values.

We next characterized anti-SARS-CoV-2 cellular responses by using an EliSPOT that quantifies IFN-γ following wild-type SARS-CoV-2 S1-peptide stimulation of PBMCs. There was a significant increase in IFN-γ spots per 10^6^ PBMCs incubated with S1-peptides at two months compared to pre-vaccination (*p*=0.014, **Figure 5A**) with 38% of KTRs developing an IFN-γ cellular response. When stratified by anti-spike antibody status, there was no difference in the probability of developing a cellular response in antibody-positive vs antibody-negative KTRs (28% vs 41%, p=0.911, **Figure 5B**). We also found no difference in the number of IFN-γ spots per 10^6^ PBMCs post-vaccination in antibody-positive vs antibody-negative KTRs (*p*=0.841), and no correlation between IFN-γ spots post-vaccination and anti-wild-type spike antibody MFIs, (r=0.106, **Figure S5A**) anti-S1 antibody MFIs (r=0.115, **Figure S5B**), anti-RBD antibody MFIs (r=0.092, **Figure S5C**) or anti-wild-type neutralizing antibody concentrations (r=0.222, **Figure S5D**).

Flow cytometry analysis of circulating T-cell subsets in all KTRs (**Figure S2**) showed a decrease in the percentage of total T-cells (*p*=0.006, **Figure 5C**) and CD4^+^ T-cells (*p*=0.013, **Figure 5D**) but an increase in the percentage of CD8^+^ T-cells (*p*=0.005, **Figure 5E**). Analysis of CD4^+^ T-cell subsets showed an increase in the percentage of regulatory T cells post-vaccination (*p*=0.020, **Figure 5F**), but no change in the percentage of T follicular helper (*p*=0.999, **Figure S6A**), T follicular regulatory (*p*=0.132, **Figure S6B**), CD4^+^ naïve (*p*=0.426, **Figure S6C**), CD4^+^effector memory (*p*=0.357, **Figure S6D**), CD4^+^central memory (*p*=0.092, **Figure S6E**) or CD4^+^effector memory RA (TEMRA) T-cells (*p*=0.054, **Figure S6F**) post-vaccination. Stratified analysis of CD4^+^ T-cell subsets by anti-spike antibody status showed no change in the percentage of T-follicular helper cells in either group (**Figure 5G**) but showed an increase in the percentage of T-follicular regulatory cells in antibody-negative KTRs (*p*=0.049, **Figure S6G**). Analysis of CD8^+^ T-cell subsets showed a decrease in the percentage of CD8^+^ naïve T-cells (*p*=0.021, **Figure S6H**) and an increase in the percentage of CD8^+^ effector memory T-cells (*p*=0.027, **Figure S6I**), but no changes in the percentages of CD8^+^ central memory (*p*=0.931, **Figure S6J**) or CD8^+^ TEMRA T-cells (*p*=0.302, **Figure S6K**).

### Characteristics Associated With Developing Antibody and Cellular Immune Responses

When evaluating factors associated with antiviral antibody responses, univariate analysis showed that female gender, non-mycophenolate-containing regimens, and steroid-free maintenance regimens were associated with higher odds of developing anti-spike antibodies post-vaccination. After adjustment for age, gender, months post-transplantation, mycophenolate and steroid use in a multivariable logistic regression model, only non-mycophenolate-based regimens and steroid-free regimens remained associated with higher odds of developing anti-spike antibodies (**Table 2**).

**Table 2:** Univariate and multivariate logistic regression analysis of characteristics associated with the development of anti-spike antibodies following vaccination in kidney transplant recipients.

Independent variables	Unadjusted OR (95% CI)	Adjusted OR (95% CI)
Female vs male	4.22 (1.36-14.15)	3.50 (0.97-13.63)
Age ≥60 vs <60 years	0.85 (0.28-2.66)	0.44 (0.10-1.82)
Deceased vs living donor	0.38 (0.12-1.17)	–
Months from transplant: <48 vs ≥48	0.75 (0.23-2.31)	0.72 (0.18-2.75)
Basiliximab vs ATG induction	1.49 (0.43-5.37)	–
Belatacept-based vs tacrolimus-based regimen	0.36 (0.07-1.38)	–
Steroid-free vs steroid-maintenance regimen	5.07 (1.44-19.82)	4.84 (1.13-23.52)
Non-MMF-based vs MMF-based regimen	4.13 (1.15-16.21)	5.38 (1.10-30.69)
MMF daily dose: >1 g vs ≤1 g	0.58 (0.08-2.83)	–
Tacrolimus trough: ≥6 vs <6 ng/mL	0.36 (0.07-1.38)	–

ATG, Antithymocyte globulin; MMF, mycophenolate.

When evaluating factors associated with cellular immune responses, we found no correlation between change in IFN-γ spots from baseline to month two and tacrolimus trough levels (r=0.041), mycophenolate dose (r=-0.230), age (r=0.077) or months post-transplantation (r=0.013). There was also no significant difference in the change in IFN-γ spots from baseline to month two between patients on tacrolimus vs belatacept (*p*=0.844), mycophenolate-based vs non-mycophenolate-based regimens (*p*=0.294) and steroid-maintenance vs steroid-free regimens (*p*=0.140).

### Adverse Events and SARS-CoV-2 Infections Following Vaccination

No patients experienced severe adverse events post-vaccination. Two patients developed SARS-CoV-2 infection during follow-up, both of whom were on belatacept and neither of whom had anti-spike antibodies post-vaccination prior to infection (**Table S2**).

## Discussion

In this study, we comprehensively evaluate allograft function, alloimmune and anti-viral responses after SARS-CoV-2 mRNA vaccination in KTRs. Since serum creatinine (30–32) and proteinuria (33) are not sensitive enough to exclude allograft rejection, we performed additional non-invasive monitoring for rejection using *de novo* DSAs by single-antigen bead platform, ddcfDNA, 5-gene PGBEP and urinary *CXCL9* mRNA by CRISPR-Cas13 platform.

No KTRs in our cohort developed *de novo* DSAs or an increase in pre-existing DSA MFIs post-vaccination. There was only one episode of rejection in a high-risk patient with a previous history of rejection and recent conversion to belatacept. The patient did not develop *de novo* DSAs, anti-spike antibodies or an increase in IFN-γ spots post-vaccination, suggesting that the rejection episode was likely unrelated to vaccination. The low risk of clinical rejection is consistent with what has been described prior in a study of 136 KTRs, none of whom developed clinical rejection after SAR-CoV-2 vaccination, although no specific monitoring for rejection was reported in the study beyond what is routinely done clinically (15). In addition, a study of 741 solid organ transplant recipients that surveyed patients 7 days after the second vaccine dose reported only one episode of rejection, but this data was collected by patient report and at a short follow-up duration (16). Another study of 148 KTRs who received two doses of mRNA-1273 found that there were no development of DSA two weeks after the second vaccine dose (13). While consistent with our findings, our study adds to the literature by having a predominantly BNT162b2-vaccinated KTR cohort, longitudinal monitoring for rejection, longer follow-up than prior studies and DSA measurement at a longer follow-up period of 3 months.

The rejection event in our cohort was detected by an elevation in serum creatinine and urinary *CXCL9* mRNA levels. *CXCL9* is a major chemokine that attracts immune cells to allografts during rejection and has been shown in a multicenter study to be an important non-invasive urinary biomarker of rejection (34). A recent study showed that elevated urinary *CXCL9* mRNA levels detected by CRISPR-Cas13 platform had 93% sensitivity for allograft rejection in KTRs (27). However, our study was underpowered to evaluate the utility of urinary *CXCL9* mRNA levels as a rejection marker and we did not perform surveillance biopsies to evaluate if urinary *CXCL9* mRNA levels are able to detect subclinical rejection.

Plasma ddcfDNA levels, expressed as a percentage of total cell-free DNA, have been shown to be a sensitive marker of alloantibody-mediated rejection, in KTRs (22). However, ddcfDNA levels may be less sensitive for the detection of T-cell mediated rejection (22), which is likely the reason it was not elevated in the patient who developed cellular rejection. The PBGEP (AlloMAP-Kidney) score derived from the expression of five candidate genes has been recently described as a marker of immune quiescence in KTRs (24). For discrimination between rejection and immune quiescence, it has an area under receiver operating characteristic curve of 0.78 and 0.89 as a standalone test and when combined with ddcfDNA, respectively (24). The PBGEP score remained stable following vaccination in our cohort, including in the patient who developed cellular rejection. Further studies are needed to validate the PBGEP score in KTRs including potentially adding other genes to the panel to improve its sensitivity.

This very low rate of clinical acute rejection, in combination with lack of *de novo* DSA development and stable levels of creatinine, proteinuria, and ddcfDNA levels suggests that SARS-CoV-2 mRNA vaccination is not associated with significant alloimmune responses in KTRs within three months of vaccination. We cannot, however, rule out the occurrence of subclinical rejection since surveillance biopsies were not performed.

In terms of anti-viral responses, we confirmed the low percentage of KTRs who develop anti-spike antibodies post-vaccination described previously (2–4, 8–10, 13, 15, 35–39). We then assessed antibody neutralization capacity using a SVNT, the results of which have been shown to be highly correlated with that of conventional live virus neutralization test (R^2^ = 0.8591) (40). We found that after two mRNA vaccine doses, there was a trend towards a lower proportion of KTRs having neutralizing responses against the Delta variant compared to wild-type SARS-CoV-2, but this did not meet statistical significance. An important limitation of SVNT is that it only measures RBD-ACE2 interactions, neutralizing antibodies directed against non-RBD regions of the spike protein may not be detected using this assay.

We found that KTRs on mycophenolate-containing regimens were less likely to develop anti-spike antibodies post-vaccination, similar to previous reports in KTRs following influenza (41, 42) and SARS-CoV-2 vaccination (2, 15, 37, 38, 43, 44). This is consistent with mycophenolate’s known effect of reducing antibody production in response to foreign antigens mediated by inosine monophosphate dehydrogenase inhibition resulting in diminished B-cell proliferation (45, 46). KTRs on steroid-maintenance regimens were also less likely to develop anti-spike antibodies post-vaccination, similar to previous reports (15, 37).

We found that 38% of KTRs had a cellular immune response by IFN-γ EliSPOT post-vaccination, which is similar to the 30-58% cellular response rates reported in other studies (8, 10, 13, 47). In combination with seroconversion data discussed above, this suggests that KTRs have reduced responses to mRNA vaccination in both B and T-cell arms of the adaptive immune system. Interestingly, we did not find a correlation between immunosuppression regimen characteristics and the magnitude of IFN-γ EliSPOT response in KTRs. While other studies have reported similar findings (9, 10) one study reported that diabetes mellitus, lymphopenia and an eGFR of <60 ml/min/1.73m^2^ were associated with lower probability of developing a cellular response (13). Importantly, we also found no correlation between the development of anti-spike antibodies and IFN-γ EliSPOT cellular responses, as has been reported previously (10, 13). This suggests that the presence or absence of antibody responses post-vaccination cannot be used to predict whether KTRs have developed cellular responses or not. Both antibody and cellular responses should be measured to fully evaluate anti-viral responses in this high-risk population.

Immunophenotyping of circulating lymphocytes showed a significant increase in the percentage of IgM+ B-cells and IgM+ plasma cells after vaccination in anti-spike antibody positive KTRs. The percentage of T follicular regulatory cells increased after vaccination in anti-spike antibody negative KTRs post-vaccination, consistent with their function in regulating antibody responses (48). We also found a decrease in the percentage of CD8^+^ naïve T cells and an increase in the percentage of CD8^+^ effector memory T cells after vaccination, suggesting a possible effect of SARS-CoV-2 vaccination on effector T cell function in KTRs (49, 50). Despite these changes, using immune phenotyping of circulating lymphocytes alone is insufficient to distinguish KTRs who will or will not develop anti-spike antibodies following vaccination. A limitation of our immunophenotyping is that the changes noted (e.g. increase in the percentage of IgM+ B-cells) were for the total pool of the analyzed immune cells, and not antigen-specific cells from each subset. Assessment of germinal center responses in the draining lymph nodes of vaccinated KTRs is likely to be more informative compared to immunophenotyping of circulating lymphocytes with regards to predicting antibody responses to vaccination (51).

Only two patients (3%) developed symptomatic SARS-CoV-2 infections during follow-up. This rate of breakthrough infection is similar to what has been reported previously in KTRs (15, 39, 52, 53), but is higher than what has been reported in healthy individuals of 0.08-0.4% (54, 55). Neither of the two patients had anti-spike antibodies prior to infection. This is similar to what has been reported in other studies of breakthrough infections in KTRs (53, 56), and may suggest a potentially increased susceptibility to infection in KTRs who do not develop antibodies post-vaccination. The effect of antibody and cellular immune responses on the risk and severity of SARS-CoV-2 infection in KTRs needs to be explored in future studies.

Our study has several limitations including the relatively small sample size, predominantly BNT162b2-vaccinated cohort, the lack of a matched unvaccinated KTR control group for comparison, and inability to diagnose subclinical rejection since no surveillance biopsies were performed. Despite these limitations, we were able to perform a detailed and granular characterization of both anti-viral and alloimmune responses post-vaccination.

In summary, we found that SARS-CoV-2 mRNA vaccination in KTRs was safe and not associated with significant alloimmune risks or decline in allograft function. Despite diminished anti-viral antibody and cellular responses, we found that the absolute risk of developing symptomatic SARS-CoV-2 infection post-vaccination was higher, albeit with very small numbers, than previously reported in immunocompetent individuals (54, 55). Further studies are needed to determine the degree of clinical protection against SARS-CoV-2 infection gained by the cellular and antibody immune responses generated and the long-term efficacy of mRNA vaccines in KTRs to inform future vaccination strategies, including higher doses of vaccines, additional booster doses of the same vaccine or a combination of vaccines (43, 57–61).

## Data Availability Statement

The raw data supporting the conclusions of this article will be made available by the authors, without undue reservation.

## Ethics Statement

The studies involving human participants were reviewed and approved by Mass General Brigham Institutional Review Board (Protocol#: 2021P000043). The patients/participants provided their written informed consent to participate in this study.

## Author Contributions

Conceptualization, AAJ, RG, TB, OE, AA, JC, CK, JA, and LR. Data curation, AAJ, FH, PP, and MM. Formal analysis, AAJ, RG, TB, ZS, FH, IL, OE, BB, VP, IR, PC, and LR. Funding acquisition, AAJ, JA, and LR. Investigation, AAJ, RG, TB, ZS, IL, BB, PC, CK, and LR. Methodology, AAJ, RG, TB, IL, OE, PP, JC, BB, VP, PC, CK, JA, and LR. Project administration, AAJ, ZS, FH, IL, OE, AA, PP, MM, VP, JA, and LR. Validation, AAJ and IL. Visualization, AAJ, TB, and IR. Supervision, IL, AA, PP, JC, MM, BB, and LR. Writing-original draft, AAJ and IR. Writing-review and editing, AAJ, RG, TB, ZS, IL, VP, PC, CK, JA, and LR. All authors contributed to the article and approved the submitted version.

## Funding

The study was funded by CareDx, Inc. (Brisbane, CA) grant number 2021A008053 to LR and JA. The study was also supported in part by the Harold and Ellen Danser Endowed/Distinguished Chair in Transplantation at Massachusetts General Hospital (Boston, MA, USA). This was an investigator-initiated research project where the design and conduct of the study was determined by the investigator without influence from the funders.

## Conflict of Interest

The authors declare that the research was conducted in the absence of any commercial or financial relationships that could be construed as a potential conflict of interest.

## Publisher’s Note

All claims expressed in this article are solely those of the authors and do not necessarily represent those of their affiliated organizations, or those of the publisher, the editors and the reviewers. Any product that may be evaluated in this article, or claim that may be made by its manufacturer, is not guaranteed or endorsed by the publisher.
