# Supplementary material for: Non-Invasive Monitoring for Rejection in Kidney Transplant Recipients After SARS-CoV-2 mRNA Vaccination
Source: Front Immunol. 2022 Feb 25;13:838985. doi: 10.3389/fimmu.2022.838985 (PMC8913529; doi:10.3389/fimmu.2022.838985)
Supplement: Supplementary file 1 [file DataSheet_1.pdf]

Supplementary Material

Table of Contents:

- Supplementary tables (S1-S2): pages 2-3
- Supplementary figures (S1-S6): pages 4-9
- Supplementary materials and methods: page 10
- References: pages 11-12

Supplementary tables

Table S1. Donor-specific antibodies at the time of vaccination.

Subject ID	HLA class	HLA locus
M15	II	DQ5
M25	II	DR12
B5	II	DQ7
B16	I	A32
B19	II	DQ7

Table S2. SARS-CoV-2 infection after vaccination

Subject ID	Days between 2nd vaccine dose and COVID-19 diagnosis	Anti-spike antibody response	IFN-γ cellular response	Illness course
M16	29	Negative	Negative	Developed hypoxemia requiring hospitalization. She was treated with remdesivir and dexamethasone, which replaced her maintenance prednisone, and her mycophenolate and belatacept were held temporarily. Her hospital course was complicated by bacterial pneumonia and *Aspergillus* pneumonia. She was eventually discharged after a 39-day admission
M21	55	Negative	Positive	Only had a dry cough and did not require treatment or hospital admission.

Supplementary figures

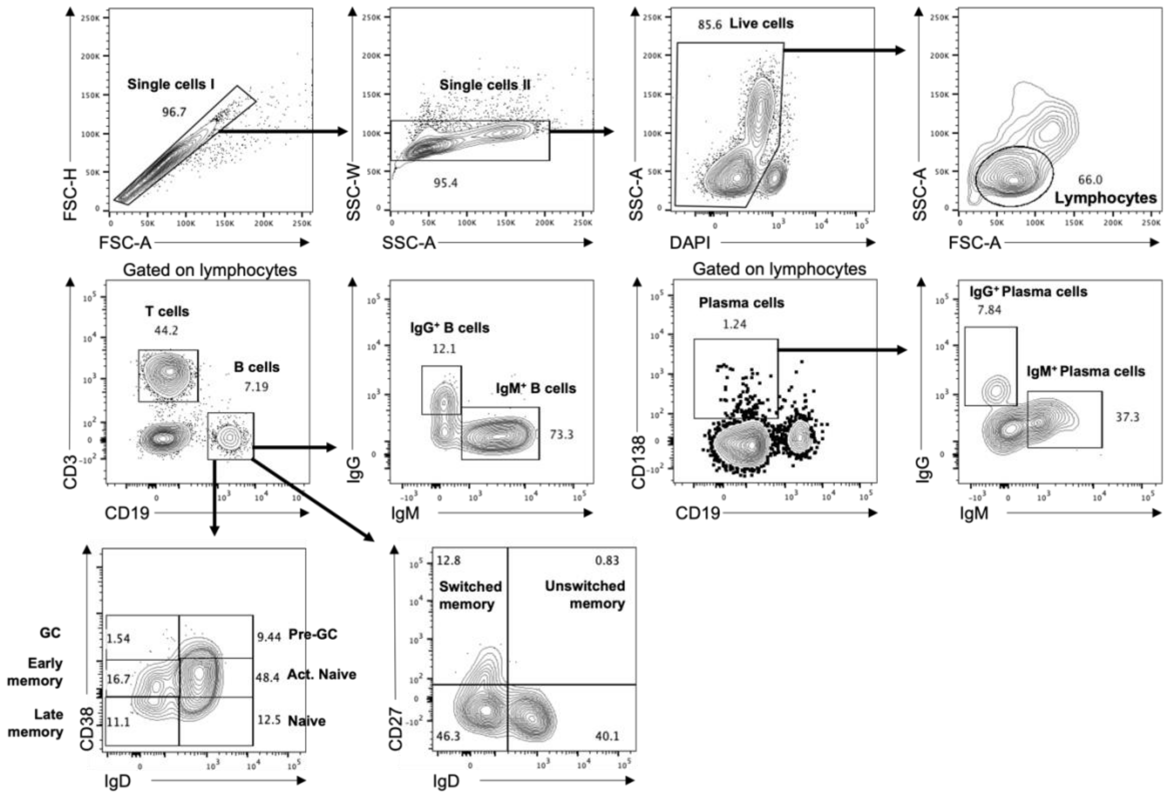

Figure S1. Flow cytometry gating strategy for B cells. Gating strategy for B cell subsets. GC: germinal center. Act. naïve: activated naïve.

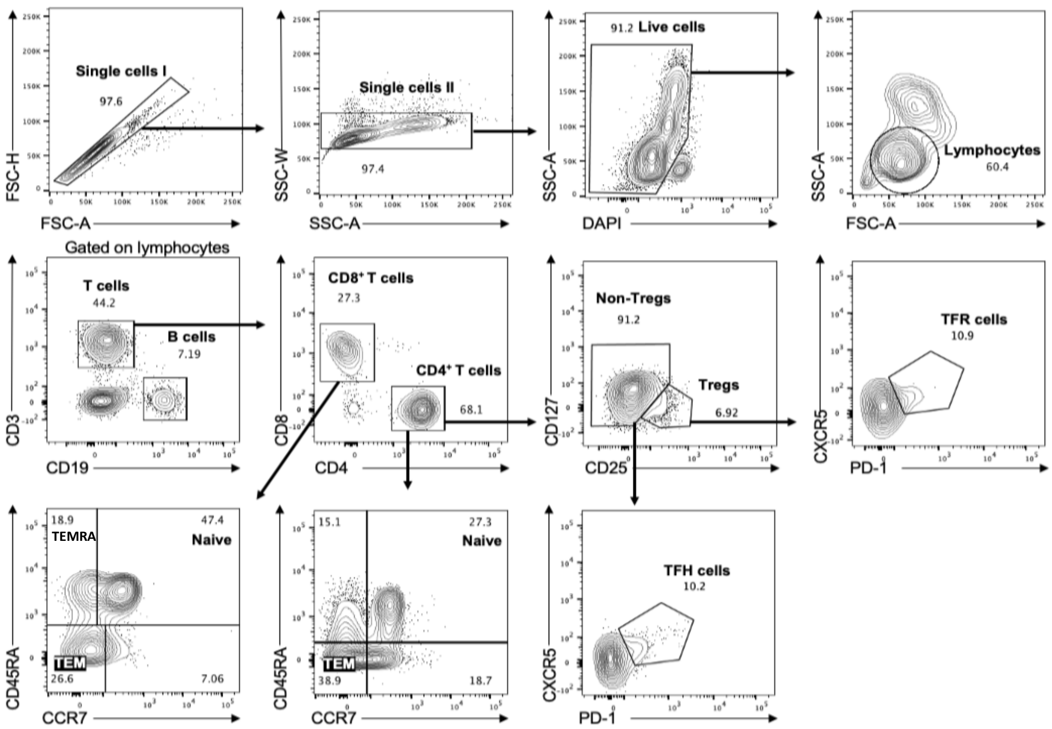

Figure S2. Flow cytometry gating strategy for T cells. Gating strategy for T cell subsets. TEM: T effector memory cells. TFH: T follicular helper cells. TFR: T follicular regulatory cells. Tregs: regulatory T cells.

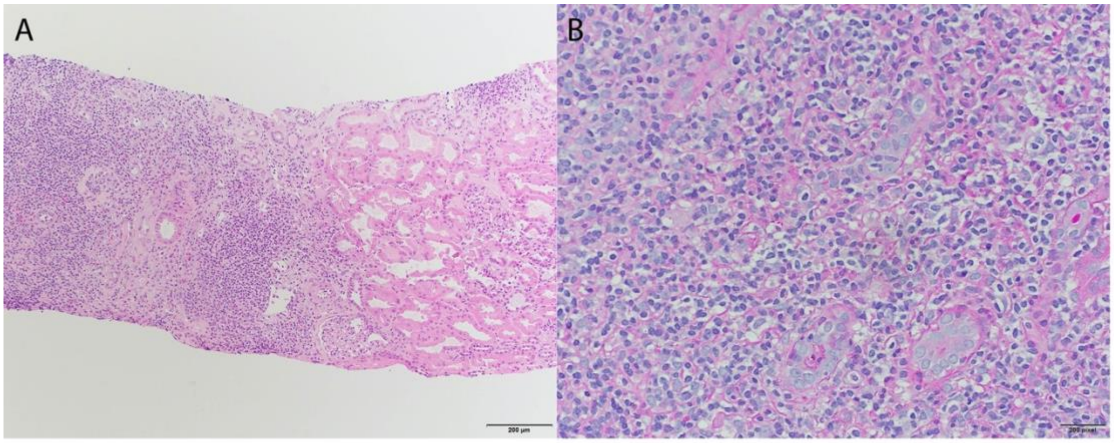

Figure S3. Acute cellular rejection in one kidney transplant recipient following SARS-CoV-2 mRNA vaccination. **(A)** Low power magnification images of kidney allograft biopsy showing foci of dense interstitial inflammation (H&E 100x). **(B)** The infiltrate showed lymphocytes with numerous eosinophils, associated with severe tubulitis and many foci of tubular basement membrane destruction, consistent with acute T cell mediated rejection (Banff type IB; PAS, 400x).

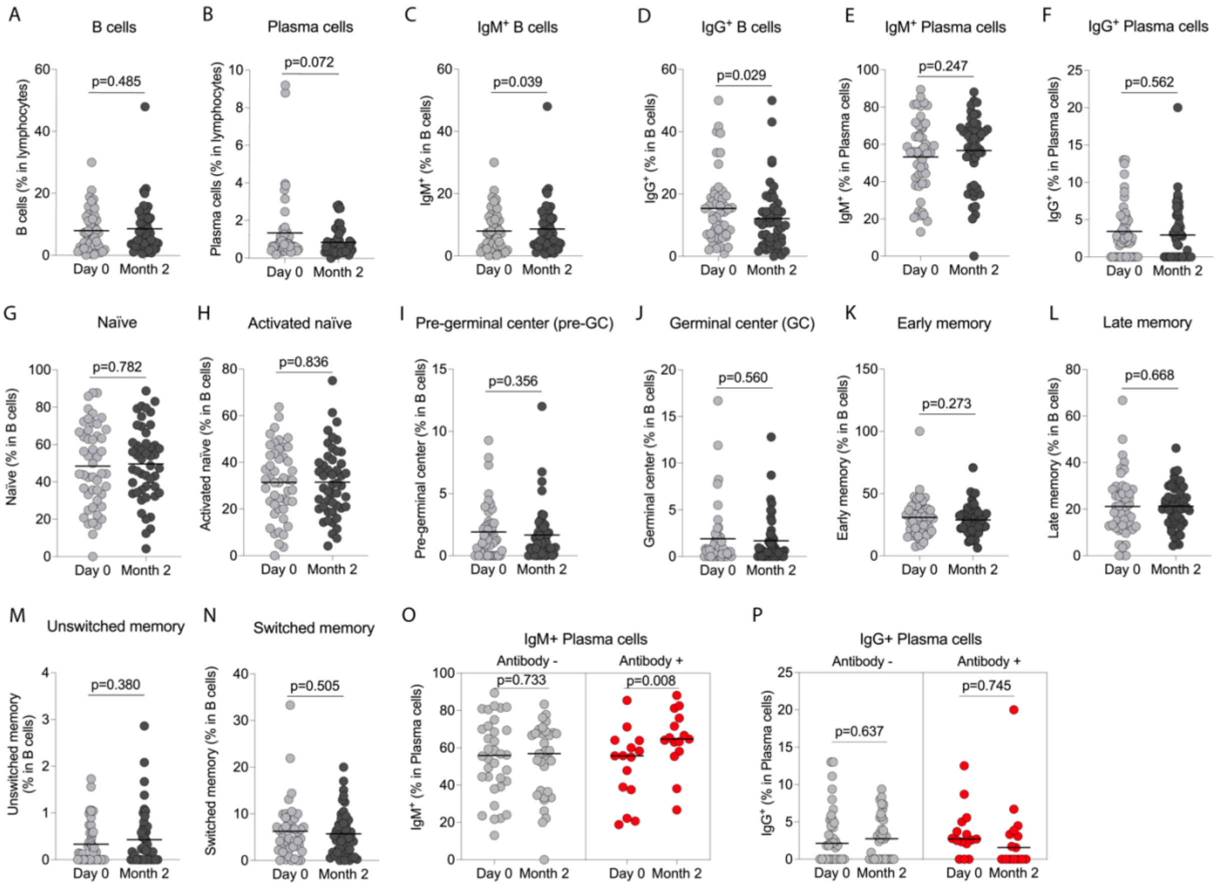

Figure S4. Humoral immune responses following SARS-CoV-2 mRNA vaccination in kidney transplant recipients (KTRs). Percentage of circulating (**A**) B cells, **(B)** plasma cells, **(C)** IgM$^+$ B cells**, (D)** IgG$^+$ B cells**, (E)** IgM$^+$ plasma cells, **(F)** IgG$^+$ plasma cells, **(G)** naïve B cells, **(H)** activated naïve B cells, **(I)** pre-germinal center B cells, **(J)** germinal center B cells, **(K)** early memory B cells, **(L)** late memory B cells, **(M)** unswitched memory B cells, **(N)** and switched memory B cells in KTRs following vaccination. Percentage of circulating **(O)** IgM$^+$ and **(P)** IgG$^+$ plasma cells in KTRs who developed anti-spike antibodies compared to KTRs who did not develop anti-spike antibodies. (A-P) Statistics by paired t test (n=49).

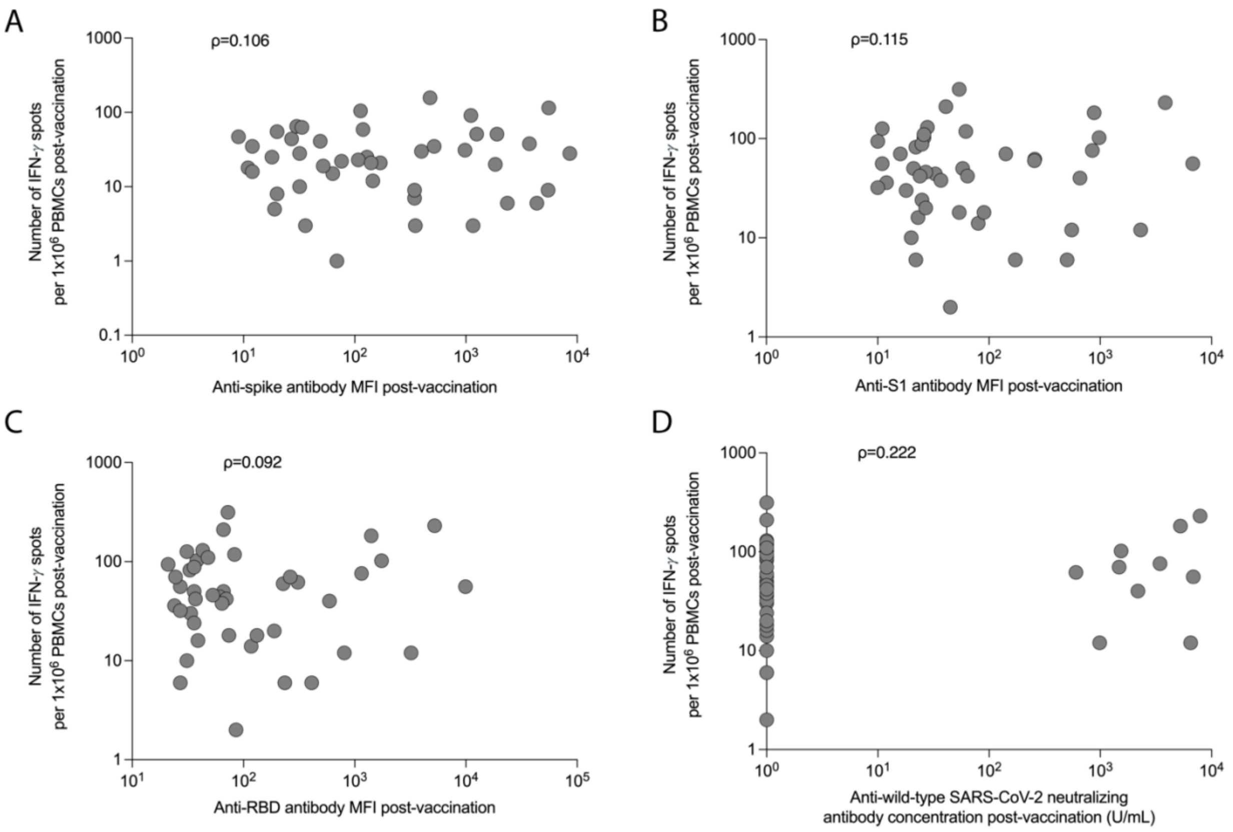

Figure S5. Associations between cellular and humoral immune responses following SARS-CoV-2 mRNA vaccination in kidney transplant recipients (KTRs). Spearman correlation between interferon gamma spots and **A)** anti-wild-type SARS-CoV-2 spike antibody median fluorescent intensities (MFIs), **B)** anti-S1 antibody MFIs, **C)** anti-receptor binding domain (RBD) antibody MFIs, and **D)** anti-wild type SARS-CoV-2 neutralizing antibody concentrations.

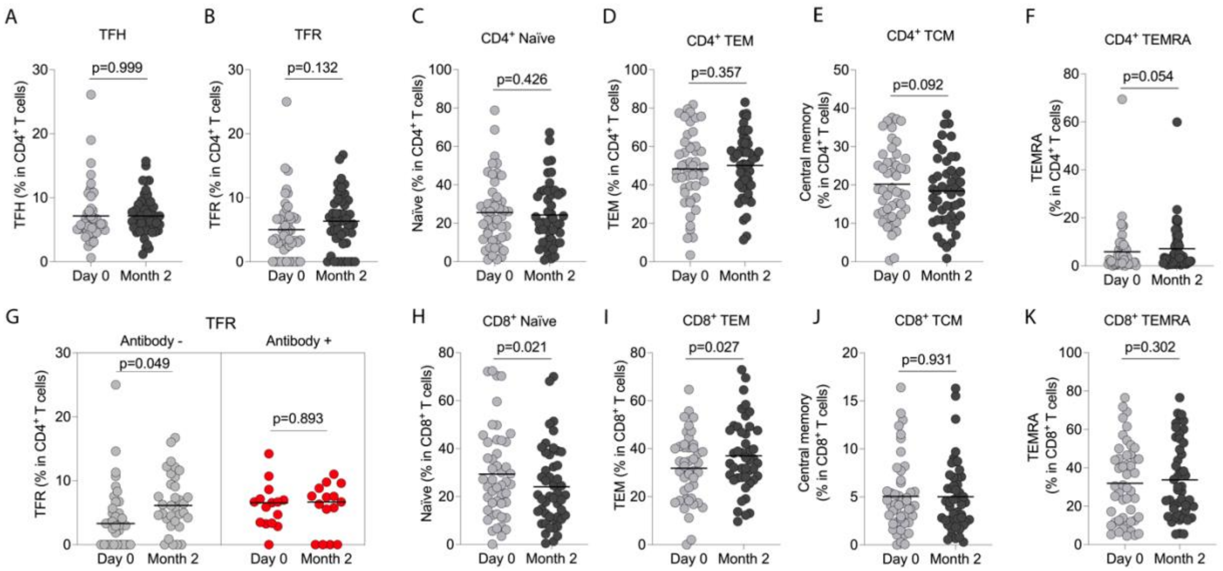

Figure S6. Cellular immune responses following SARS-CoV-2 mRNA vaccination in kidney transplant recipients (KTRs). Percentage of circulating CD4^{+} **(A)** T follicular helper (TFH), naïve, **(B)** T follicular regulatory (TFR), **(C)** naïve, **(D)** effector memory (TEM), **(E)** central memory (TCM), and **(F)** effector memory RA (TEMRA) T cells in KTRs following vaccination. **(G)** Percentage of circulating T follicular regulatory (TFR) cells in KTRs following vaccination stratified by anti-spike antibody status. Percentage of circulating CD8^{+} **(H)** naïve, **(I)** TEM, **(J)** TCM and **(K)** TEMRA T cells in KTRs following vaccination. (A, C-E, H-K) Statistics by paired t test. (B, F, G) Statistics by Wilcoxon matched-pairs signed rank test (n=49).

Supplementary materials and methods

Exclusion criteria:

- Age<18 years of age
- Unstable allograft function (>20% variation in last two eGFR values measured at least one week apart)
- Cellular or antibody-mediated rejection during the previous six months
- Multi-organ transplantation
- Pregnant or lactating females
- Allergy to any component of mRNA-1273 or mRNA-BNT162b2 vaccines
- Investigational drug use within 30 days of enrollment
- Receipt of non-live vaccine with 2 weeks or live viral vaccine within 4 weeks of SARS-CoV-2 vaccination
- Acute or chronic illness at the time of vaccination which in the opinion of the investigator will alter immune response (e.g. human immunodeficiency virus infection, primary immunodeficiency disease, disseminated or untreated malignancy, or systemic infection).

References:

1. Kaminski MM, Alcantar MA, Lape IT, Greensmith R, Huske AC, Valeri JA, Marty FM, Klämbt V, Azzi J, Akalin E, et al. A CRISPR-based assay for the detection of opportunistic infections post-transplantation and for the monitoring of transplant rejection. *Nat Biomed Eng* (2020) **4**:601–609. doi:10.1038/s41551-020-0546-5
2. Wong L, Scott S, Grskovic M, Dholakia S, Woodward Robert N. Medical Diagnostic Methods The Evolution and Innovation of Donor-Derived Cell-Free DNA Testing in Transplantation. *J Med Diagn Meth* (2020) **9**: doi:10.35248/2168-9784.2020.9.302
3. Bloom RD, Bromberg JS, Poggio ED, Bunnapradist S, Langone AJ, Sood P, Matas AJ, Mehta S, Mannon RB, Sharfuddin A, et al. Cell-Free DNA and Active Rejection in Kidney Allografts. *J Am Soc Nephrol* (2017) **28**:2221–2232. doi:10.1681/ASN.2016091034
4. Stites E, Kumar D, Olaitan O, John Swanson S, Leca N, Weir M, Bromberg J, Melancon J, Agha I, Fattah H, et al. High levels of dd-cfDNA identify patients with TCMR 1A and borderline allograft rejection at elevated risk of graft injury. *Am J Transplant* (2020) **20**:2491–2498. doi:10.1111/ajt.15822
5. Akalin E, Weir MR, Bunnapradist S, Brennan DC, Delos Santos R, Langone A, Djamali A, Xu H, Jin X, Dholakia S, et al. Clinical Validation of an Immune Quiescence Gene Expression Signature in Kidney Transplantation. *Kidney360* (2021)10.34067/KID.0005062021. doi:10.34067/KID.0005062021
6. Deng MC, Eisen HJ, Mehra MR, Billingham M, Marboe CC, Berry G, Kobashigawa J, Johnson FL, Starling RC, Murali S, et al. Noninvasive Discrimination of Rejection in Cardiac Allograft Recipients Using Gene Expression Profiling. *Am J Transplant* (2006)

6:150–160. doi:10.1111/j.1600-6143.2005.01175.x

7. Christakoudi S, Runglall M, Mobillo P, Tsui T-L, Duff C, Domingo-Vila C, Kamra Y, Delaney F, Montero R, Spiridou A, et al. Development of a multivariable gene-expression signature targeting T-cell-mediated rejection in peripheral blood of kidney transplant recipients validated in cross-sectional and longitudinal samples. *EBioMedicine* (2019) **41**:571–583. doi:10.1016/j.ebiom.2019.01.060

8. Ni L, Ye F, Cheng M-L, Feng Y, Deng Y-Q, Zhao H, Wei P, Ge J, Gou M, Li X, et al. Detection of SARS-CoV-2-Specific Humoral and Cellular Immunity in COVID-19 Convalescent Individuals. *Immunity* (2020) **52**:971-977.e3. doi:10.1016/j.immuni.2020.04.023

9. Janetzki S, Price L, Schroeder H, Britten CM, Welters MJP, Hoos A. Guidelines for the automated evaluation of Elispot assays. *Nat Protoc* (2015) **10**:1098–115. doi:10.1038/nprot.2015.068

10. Cucchiari D, Egri N, Bodro M, Herrera S, Del Risco-Zevallos J, Casals-Urquiza J, Cofan F, Moreno A, Rovira J, Banon-Maneus E, et al. Cellular and humoral response after mRNA-1273 SARS-CoV-2 vaccine in kidney transplant recipients. *Am J Transplant* (2021) doi:10.1111/ajt.16701
